# Identification of a hub gene VCL for atherosclerotic plaques and discovery of potential therapeutic targets by molecular docking

**DOI:** 10.1186/s12920-024-01815-9

**Published:** 2024-01-29

**Authors:** Chong Wu, Wei Li, Panfeng Li, Xiaoyang Niu

**Affiliations:** 1 The Fifth Clinical Medical College of Henan University of Chinese Medicine (Zhengzhou People’s Hospital), Zhengzhou, 450046 China; 2 Clinical Laboratory, Qingdao Women and Children’s Hospital Affiliated, Qingdao University, Qingdao, 266034 China; 3 Department of Vascular Surgery, Heart Center of Henan Provincial People’s Hospital, Fuwai Central China Cardiovascular Hospital, Central China Fuwai Hospital of Zhengzhou University, Zhengzhou, 450000 China; 4 Department of Vascular Surgery, The Fifth Affiliated Hospital of Zhengzhou University, Zhengzhou, 450000 China

**Keywords:** Atherosclerosis, Plaque, VCL, Hub gene, Molecular docking, Therapeutic target

## Abstract

**Background:**

Atherosclerosis (AS) is a pathology factor for cardiovascular diseases and instability of atherosclerotic plaques contributes to acute coronary events. This study identified a hub gene VCL for atherosclerotic plaques and discovered its potential therapeutic targets for atherosclerotic plaques.

**Methods:**

Differential expressed genes (DEGs) were screened between unstable and stable plaques from GSE120521 dataset and then used for construction of a protein-protein interactions (PPI) network. Through topological analysis, hub genes were identified within this PPI network, followed by construction of a diagnostic model. GSE41571 dataset was utilized to validate the diagnostic model. A key hub gene was identified and its association with immune characteristics and pathways were further investigated. Molecular docking and molecular dynamics (MD) simulation were employed to discover potential therapeutic targets.

**Results:**

According to the PPI network, 3 tightly connected protein clusters were found. Topological analysis identified the top 5 hub genes, Vinculin (VCL), Dystrophin (DMD), Actin alpha 2 (ACTA2), Filamin A (FLNA), and transgelin (TAGLN). Among these hub genes, VCL had the highest diagnostic value. VCL was selected for further analysis and we found that VCL was negatively correlated with immune score and AS-related inflammatory pathways. Next, we identified 408 genes that were highly correlated with VCL and determined potential drug candidates. The results from molecular docking and MD simulation showed compound DB07117 combined with VCL protein stably, the binding energy is -7.7 kcal/mol, indicating that compound DB07117 was a potential inhibitor of VCL protein.

**Conclusion:**

This study identified VCL as a key gene for atherosclerotic plaques and provides a potential therapeutic target of VCL for the treatment of atherosclerotic plaques.

**Supplementary Information:**

The online version contains supplementary material available at 10.1186/s12920-024-01815-9.

## Introduction

Atherosclerosis (AS), a chronic disorder, is the major underlying contributor involved in the development of most cardiovascular diseases. AS is manifested by plaque formation due to the accumulation of low-density lipoprotein cholesterol and fibrous substances in the sub-endothelial intimal layer of arteries, leading to stenosis that induces blood supply obstruction [1, 2]. Nowadays, people in developing countries have the biggest burden of AS. Women and younger people have become more affected by AS [3]. It has been estimated that the global prevalence of carotid plaque is 21.1%, accounting for 81,576 million affected cases and a percentage change of 58.97% from 2000 [4]. Plaque instability has been acknowledged as a risk factor that has been received considerable attention [5, 6]. The disruption of a balance between instability and healing of a plaque contributes to acute coronary events such as myocardial infarctions and ischemic strokes [5]. At present, although many efforts have been made to reveal the mechanism of plaque instability and develop therapeutic therapies, the pathogenic mechanisms are complex and there are no effective drugs to reduce acute coronary events. Therefore, effective strategies are needed to develop innovative biomarkers for management of atherosclerotic plaques.

High-throughput profiling has been used to screen differential expressed genes (DEGs) between atherosclerotic plaques and normal tissues [7]. Recently, novel single-cell technologies have been utilized to decipher the heterogeneity of atherosclerotic plaques and facilitate understanding of immune characteristics under atherosclerosis [8]. With the help of comprehensive analyses, potential biomarkers can be identified using data from sequencing data. A protein-protein interactions (PPI) network can be capable of predicting functionally orthologous proteins within clusters [9]. Hub genes are genes with high degree in the PPI network, indicating the presence of numerous communicating genes in the gene network [10]. Molecular docking is a computational approach forecast binding modes between small compounds or macromolecules and receptors, which is able to predict molecular interactions [11]. Molecular dynamics (MD) simulation further offers a detail complementary association with molecular docking by calculating interaction energies [12]. Although previous studies have identified potential biomarkers involved in the progression of unstable plaques or revealed molecular mechanisms of drugs for AS [13–15], hub genes for AS and their potential therapeutic targets should be further developed.

In the current study, our differentially expressed genes in stable and unstable plaques were determined by differential expression analysis. The hub genes in atherosclerosis were determined by PPI network and molecular docking analysis. Our study aims to reveal the gene network regulated by hub genes and the drug-gene-therapeutic network in atherosclerosis, which will provide a scientific basis for the molecular mechanism of atherosclerosis and the therapeutic network to provide a scientific basis.

## Materials and methods

### Data collection

We downloaded the atherosclerosis (AS) data of GSE120521 and GSE41571 from the Gene-Expression Omnibus (GEO, https://www.ncbi.nlm.nih.gov/geo/) database. GSE120521 dataset includes 4 unstable plaque samples and 4 stable plaque samples. GSE41571 dataset contains 5 unstable plaque and 6 stable plaque samples. From The Comparative Toxicology Database (http://ctdbase.org/), the target genes of AS were obtained by retrieving “Carotid artery plaque” as a key word.

### Identification of DEGs

The gene expression profiles in GSE120521 dataset were used to perform differential expression analysis between unstable plaques and stable plaques using “limma” package [16]. DEGs were screened under the threshold of|log2FC| > 1 and false discovery rate (FDR) < 0.05. Next, the AS-related DEGs were selected from the intersection of DEGs and disease target genes using a Venn diagram.

### Functional enrichment analysis

Furthermore, AS-related DEGs were used for Gene Ontology (GO) and Kyoto Encyclopedia of Genes and Genomes (KEGG) enrichment analysis using “WebGestaltR” package [17]. The GO functional enrichment included biological process (BP), cellular component (CC) and molecular function (MF) categories. Candidates with FDR < 0.05 were considered as significantly enriched pathways and GO terms.

### Construction of a PPI network

AS-related DEGs were subjected to the STRING database (https://cn.string-db.org/) and a PPI network was obtained with confidence score > 0.4. Cytoscape (version 3.9.1) [18] was employed to construct a visual PPI network and identify important nodes for further analysis. Subsequently, module-based network analysis was performed using molecular complex detection (MCODE) algorithm in Cytoscape to find tightly connected protein clusters. The default settings of the MCODE plug-in were used as follows: - Include Loops: false and Degree Cutoff: 3 for Network Scoring. Node Score Cutoff: 0.4, Haircut: true, Fluff: false, K-Core: 3, and Max. Depth from Seed: 80 for Cluster Finding.

### Screening hub genes

Hub genes were screened by analysis the topological parameters using Cytoscape. Three parameters including “Betweenness Centrality”, “Closeness Centrality” and “Degree” were used to evaluate topological properties. “Betweenness Centrality” basically counts the number of shortest paths going through a node. “Closeness Centrality” is used to measure the importance of a vertex within a given complex network, which is defined as the reciprocal the sum of the distances from node to the other nodes in the network. “Degree” is defined as the number of edges of nodes. Generally, nodes with high ranking based on the “Betweenness Centrality”, “Closeness Centrality” and “Degree” can be identified as hub genes.

### Construction and validation of a diagnostic model

To verify the role of hub genes in unstable plaques and stable plaques, we compared the differences in gene expression in GSE120521 and GSE41571 datasets. Next, the support vector machine (SVM) was employed to construct diagnostic model using “e1071” package [19] in GSE120521 dataset. Receiver operating characteristic (ROC) curves with areas under the ROC curve (AUCs) were utilized to evaluate the performance of diagnostic model. To verify the performance of the diagnostic model, the ROC curve was drawn in the GSE41571 dataset. The wilcoxon test was used to determine the statistic differences in two groups. The threshold for *P*-value was set at 0.05. *P* < 0.05 was considered statistically significant for differences between the two groups.

### Relationship of VCL with immune characteristics and pathways

Marker genes for 28 immune cells were obtained from the pan-cancer study of Charoentong et al. [20], for which we assessed their immune scores via a single-sample gene set enrichment analysis (ssGSEA) [21]. Immune cells including Activated B cell, Activated CD4 T cell, Activated CD8 T cell, Central memory CD4 T cell, Central memory CD8 T cell, Effector memeory CD4 T cell, Effector memeory CD8 T cell, Gamma delta T cell, Immature B cell, Memory B cell, Regulatory T cell, T follicular helper cell, Type 1 T helper cell, Type 17 T helper cell, Type 2 T helper cell, Activated dendritic cell, CD56bright natural killer cell, CD56dim natural killer cell, Eosinophil, Immature dendritic cell, Macrophage, Mast cell, MDSC, Monocyte, Natural killer cell, Natural killer T cell, Neutrophil, Plasmacytoid dendritic cell. Meanwhile, ESTIMATE algorithm was used to estimate immune scores [22]. To evaluate the relationship between VCL and pathways, we obtained AS-related inflammatory pathways from previous published research [23], differential pathways between stable and unstable plaques, and AS-related pathways from KEGG. Spearman’s rank correlation analysis was employed to assess the correlation between VCL and immune characteristics and pathways.

### Prediction of VCL-related gene sets and potential target drugs

We selected VCL-related genes using the rcorr function embedded in Hmisc package [24], and identified genes highly related to VCL under the threshold of correlation greater than 0.9 and *P* < 0.001. The method of Peng et al. [25] was referenced to construct a target distance-based network to determine potential drugs for atherosclerotic therapy. Initially, using key genes as potential therapeutic targets is a cornerstone in the development of therapeutic agents for AS. To find potential target drugs of VCL-related genes, we calculated the proximity of drugs and AS based on the drug target pairs from the DrugBank database (https://go.drugbank.com/) and key PPI network from stringdb (threshold score is 400) [26]. S (therapeutic AS-related gene set), D (degree of related gene set nodes in PPI), and T (set of drug target genes) were given. The distance d (s,t) was the shortest path between node s and node t (where s ϵ S, the AS-related genes; t ∈ T, the drug target genes) and was calculated as the formula 1:

$$ d(S,T) = \frac{1}{{\left| T \right|}}\sum\limits_{t \in T} {mi{n_{s \in S}}} \left( {{\text{d(s}},{\text{t)}} + \omega } \right)$$

In formula 1, ω is defined as the weight of the target gene if the target gene is one of the VCL-related genes, ω = - ln(D + 1); otherwise, ω = 0. We randomly select a group of protein nodes in the network as the simulated drug target, and the number of nodes is the same as the target size (expressed as R). Next, we calculated the distance d (S, R) between these simulated drug targets and VCL-related gene set (formula 1). After 10,000 random repetitions, the simulated reference distribution was generated. The observed distance corresponding to the actual was converted to a normalized scoring using both µ_d (S, R)_ and σ_d (S, R)_, that was z value (formula 2):

$$ z\left(S,T\right)=\frac{d\left(S,T\right)-{\mu }_{d(S,R)}}{{\sigma }_{d(S,R)}}$$

At the location of concentrated distribution in drug distance, we conducted multiple hypothesis tests using the random data obtained from reference and selected candidate drugs related to VCL-related gene set with shorted distance and FDR < 0.001.

### Molecular docking

We downloaded the 3D structure of VCL from the Protein Data Bank (PDB, https://wwwl.rcsb.org/) database. Deepsite is a protein-binding site predictor that is used to predict protein active sites [27]. We predicted the coordinates of VCL protein active sites, where center_x = 37.5, center_y = 10.5, and center_z = 18.7. AutoDock Vina [28] was utilized for molecular docking. Firstly, AutoDockTools 1.5.6 [29] prepared all input files. The coordinates of the grid box (grid) in each XYZ direction were 40 Å. The Lamarckian algorithm was applied to determine the strongest binding mode of the ligand molecule with exhaustiveness of 8 and the maximum number of conformations output of 10, and the maximum allowable energy range of 3 kcal/mol. Binding energy scores less than − 7 kcal/mol were considered to be strongly binding. The results were processed using Pymol [30].

### Molecular dynamics (MD) simulation

Gromacs2022 package [31] was used to perform MD simulations of 100 nanosecond (ns) to assess the binding stability of the receptor-ligand complex. The CHARMm36 force [32] field was employed in MD simulation. The str files of the ligands were obtained using the CHARMM Common Force Field (CGenFF) program [33, 34]. The system is solvated in the dodecahedral box of TIP3P water molecules and was neutralized by adding sodium ion and chloride ion. The system concentration was 0.154 M. The structure was next subjected to steepest descent energy minimization in 5000 steps. LINCS algorithm [35] was utilized to restrain length of covalent bond and Particle-Mesh Ewald (PME) algorithm [36] was employed to determine electrostatic interactions. Thereafter, MD simulations in canonical (NVT) ensemble and isobaric-isothermal (NPT) ensemble were conducted for 100 ps maintained at 300 K temperature and pressure of 1 bar, in which the constrained atoms of the compound equilibrated the system at their initial coordinates. A 100-ns production run was performed every 2 fs. Furthermore, the root mean square deviation (RMSD) values of the ligands were calculated using the Gromacs plug-in tool [37].

## Results

### Identification of AS-related DEGs and functional enrichment analysis

**Fig. 1:**
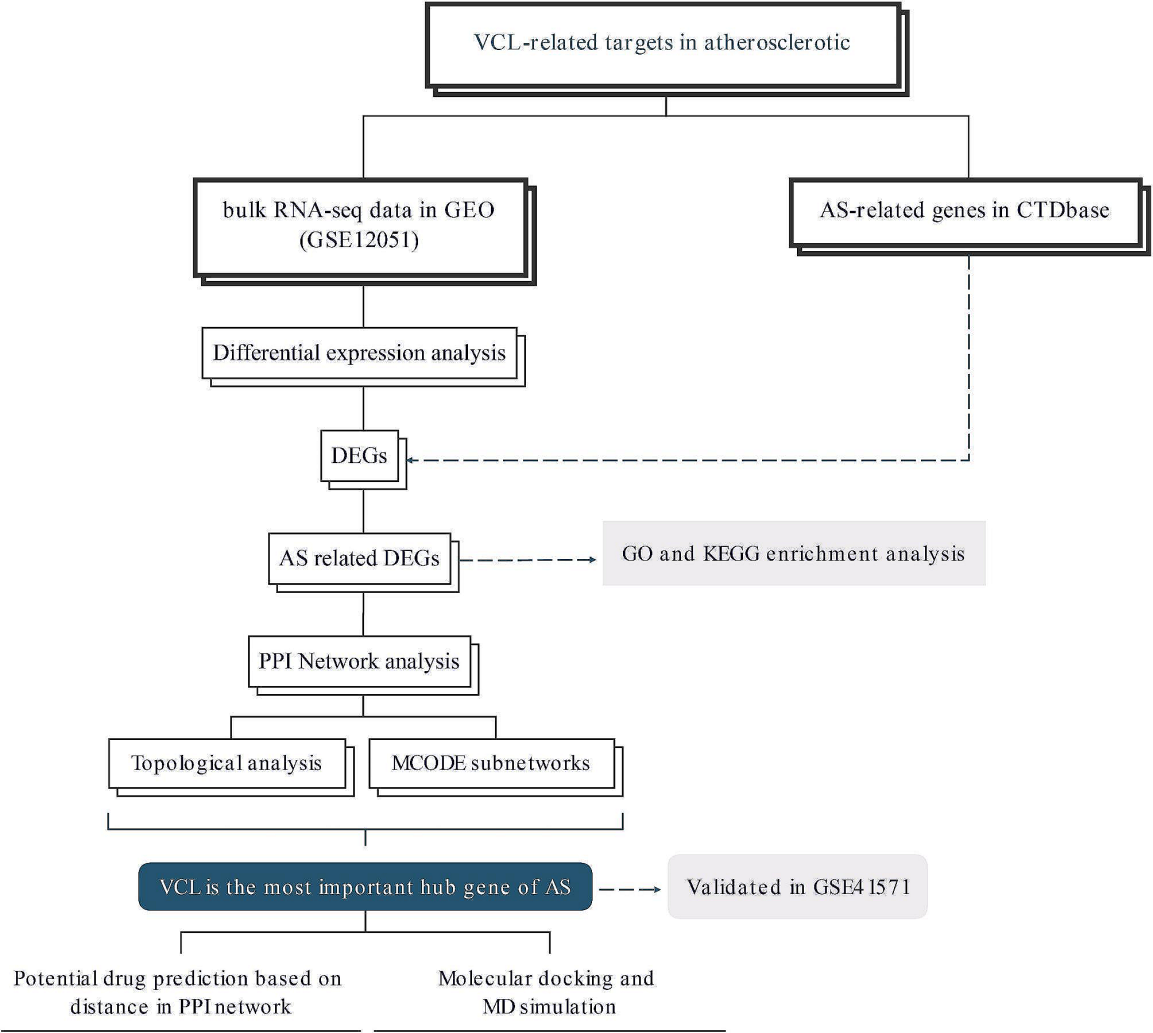
Flowchart of this research

**Fig. 2:**
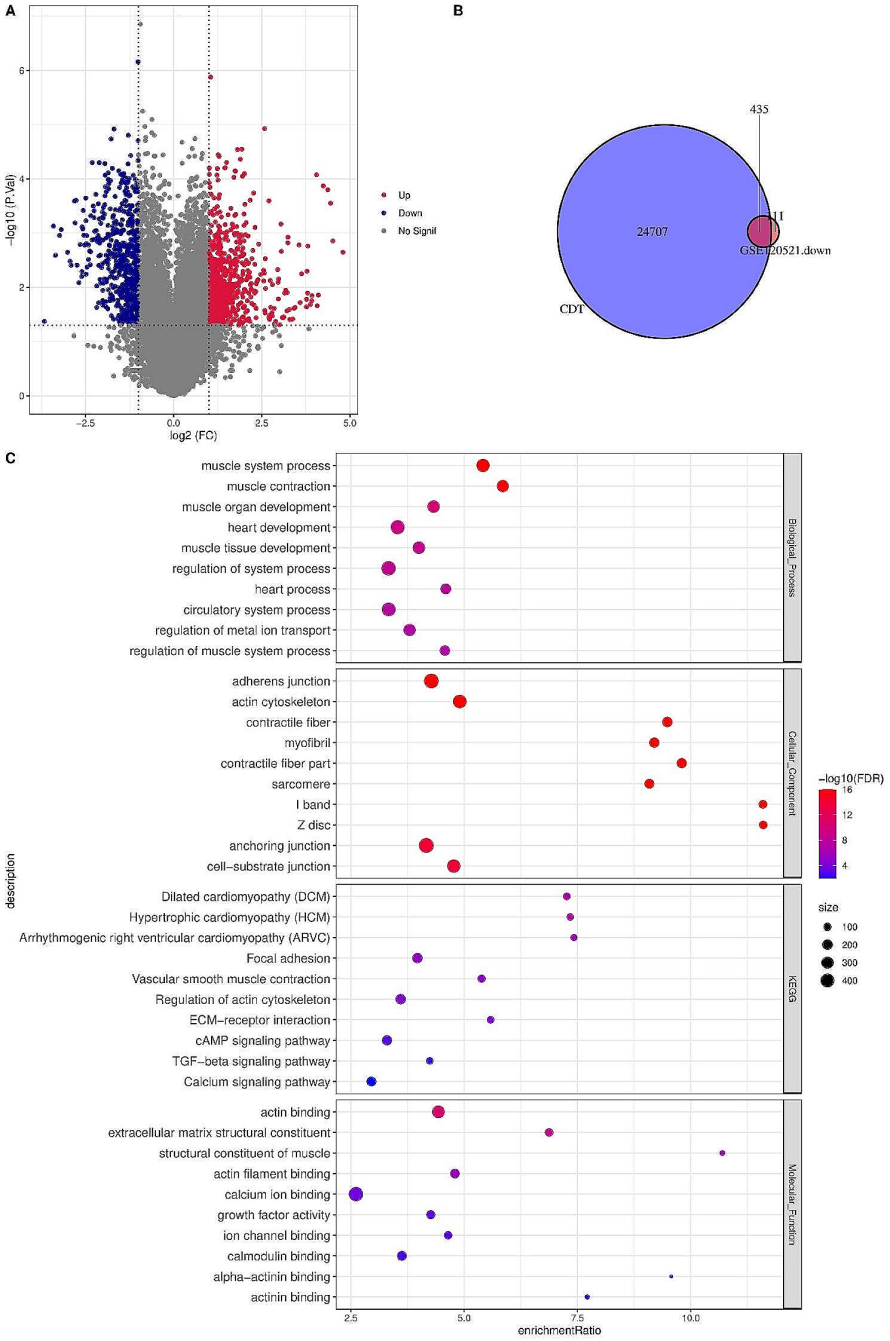
Identification of AS-related DEGs and functional enrichment analysis. A, Volcano plots displayed DEGs between unstable plaques and stable plaques in GSE120521 dataset. Red plots represent upregulated DEGs and blue ones represent downregulated DEGs. B, Venn diagram shows there were 435 DEGs at the intersection between DEGs and AS target genes. C, The top 10 most significant GO terms and KEGG pathways in the GO, KEGG enrichment analysis

The flowchart of this study was shown in Fig. 1. We screened DEGs between unstable plaques and stable plaques using differential expression analysis in GSE120521 dataset with|log2FC| > 1 and FDR < 0.05 (Fig. 2A). At the intersection between DEGs and AS target genes, 435 AS-related DEGs were identified (Fig. 2B). Under FDR < 0.05, 15 KEGG pathways, 235 BP items, 86 CC items, and 21 MF items were obtained. These AS-related DEGs were mainly enriched in focal adhesion, regulation of actin cytoskeleton, ECM-receptor interaction, cAMP signaling pathway, TGF-beta signaling pathway, and calcium signaling pathway (Fig. 2C).

### PPI network construction and hub gene determination

**Fig. 3:**
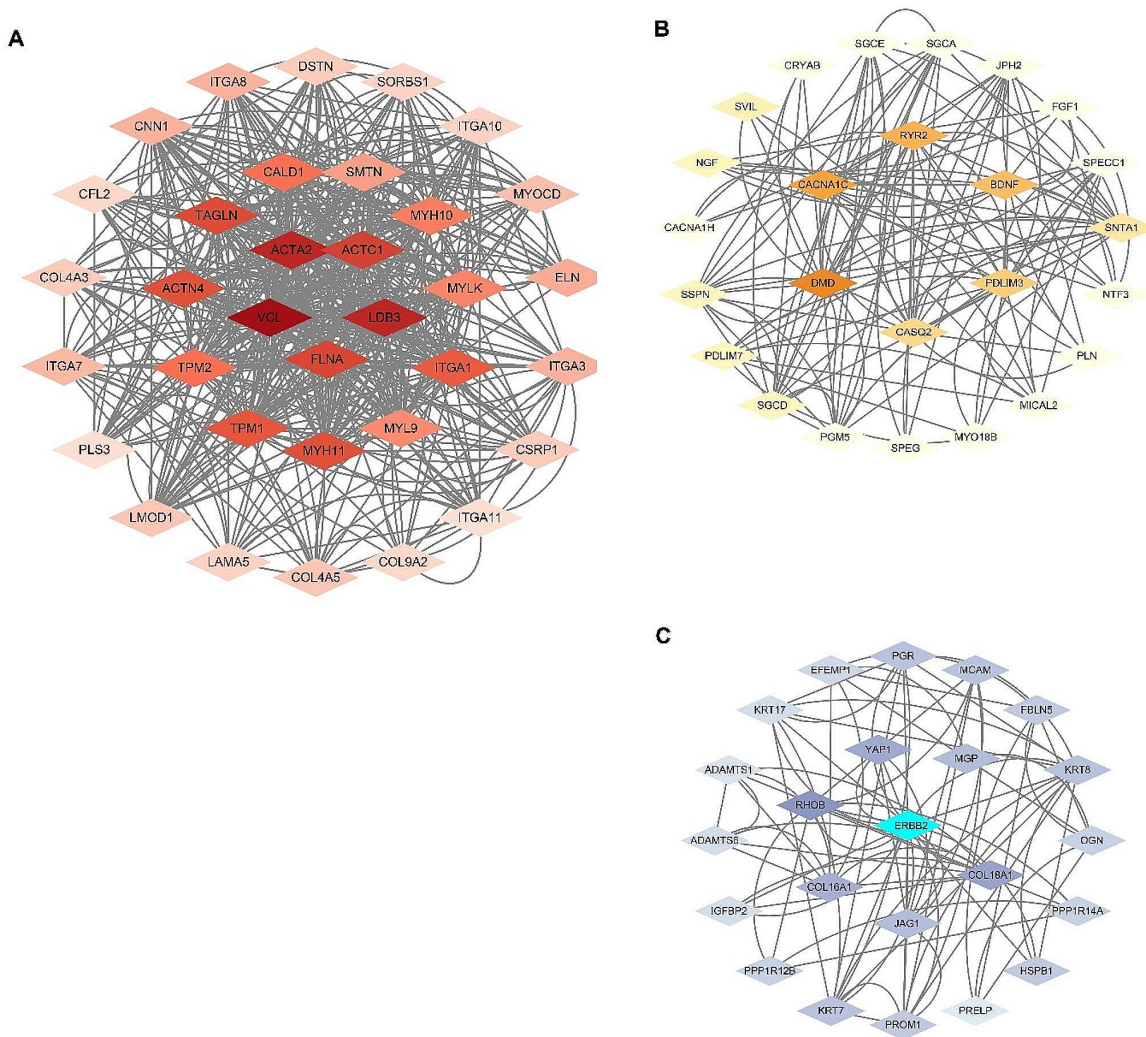
Three clusters of three 3 closely related proteins obtained by clustering analysis based on molecular complex detection (MCODE) algorithm (**A**-**C**)

AS-related DEGs were used to construct a PPI network. After removing all isolated and partially disconnected nodes, a network of targets was constructed and 364 nodes were finally retained for further analysis. Through MCODE, we found 3 tightly connected protein clusters in the target network (Fig. 3A-C). Subsequently, we performed topological analysis of the three clusters by evaluating topological parameters “Betweenness Centrality”, “Closeness Centrality” and “Degree” to determine hub genes. We selected the top 5 genes with topological parameters as hub genes. Therefore, VCL, DMD, ACTA2, FLNA, and TAGLN were the top 5 hub genes in PPI network (Table 1).

**Table 1:** Topological parameters of top 5 hub genes in PPI network

name	Degree	Betweenness Centrality	Closeness Centrality
VCL	96	0.104468007	0.427196149
DMD	66	0.071981676	0.404328018
ACTA2	86	0.06168734	0.411832947
FLNA	74	0.047363023	0.380901288
TAGLN	72	0.041099176	0.387554585

### Construction and validation of a diagnostic model

**Fig. 4:**
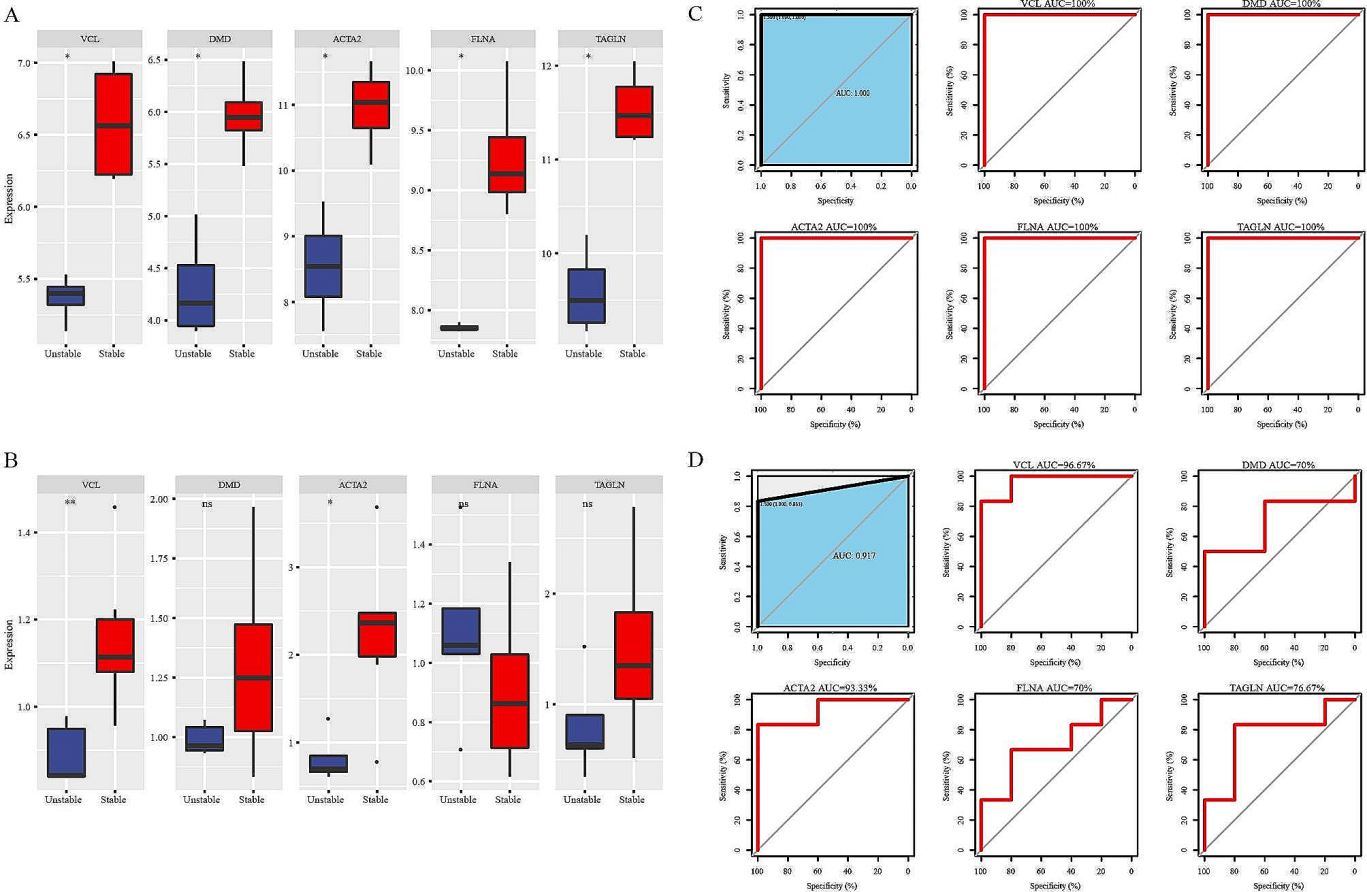
Expression levels as well as ROC curves of the diagnostic performance of the five hub genes. A-B, VCL and ACTA2 were decreased in unstable plaques in GSE120521 and GSE41571 datasets. C-D, Diagnostic model using 5 hub genes in GSE120521 and GSE41571 datasets. Ns represents P > 0.05, *P < 0.05, **P < 0.01, ***P < 0.001 and ****P < 0.0001

To further confirm the role of these 5 hub genes in plaques, we compared their expression differences between stable and unstable plaques. In GSE120521 and GSE41571 datasets, VCL and ACTA2 were significantly lower expressed in unstable plaques (*p* < 0.05) (Fig. 4A-B). Furthermore, we constructed a diagnostic model using 5 hub genes in GSE120521 and found that VCL had the highest AUC in GSE120521 dataset (Fig. 4C). Similar results were also observed in GSE41571 dataset (Fig. 4D). Accordingly, it is believed that VCL gene has research potential in atherosclerotic plaques.

### VCL is negatively correlated with immune characteristics and pathways

**Fig. 5:**
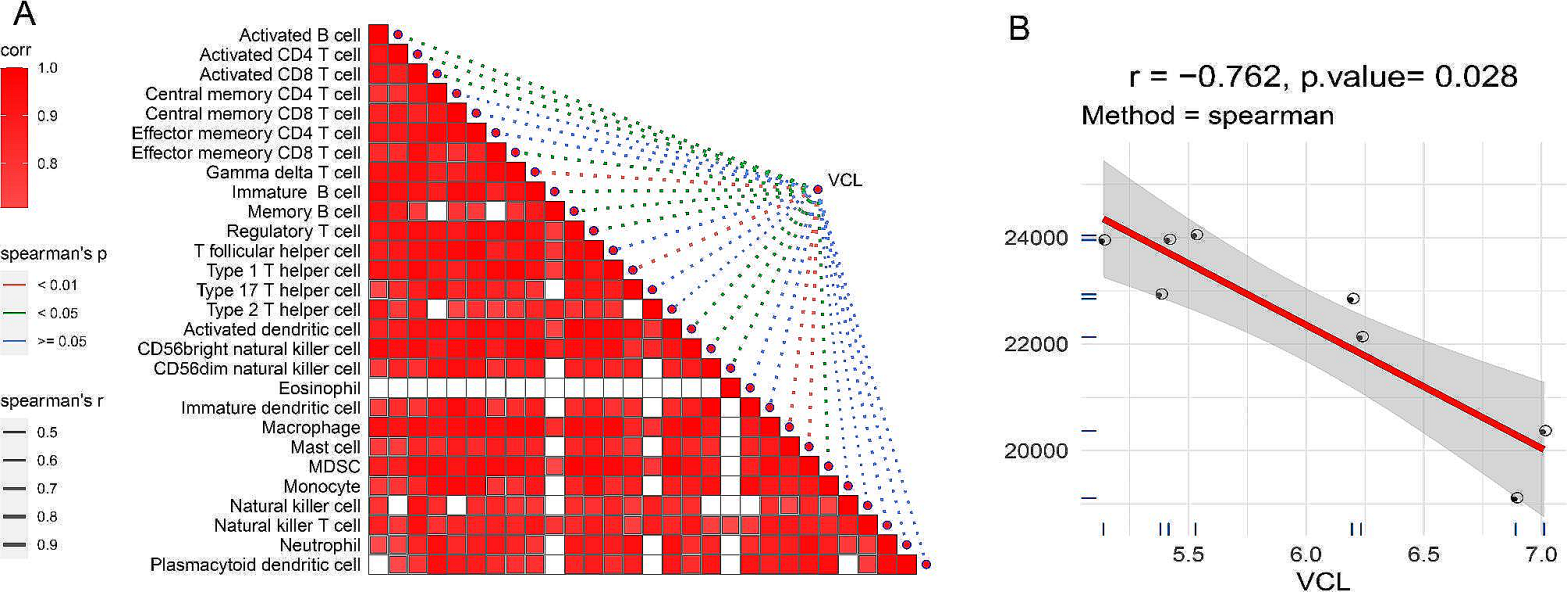
VCL is negatively correlated with immune characteristics. A, VCL is negatively correlated with most of 28 immune cells. B, VCL is negatively correlated with ImmuneScore

As shown in Fig. 5A, VCL had a significant negative correlation with most of immune cells. Meanwhile, we analyzed the association between VCL and immune score. The results showed that VCL was negatively correlated with immune score (*R*=-0.762, *P* = 0.028) (Fig. 5B). The correlation coefficients of DMD, ACTA2, FLNA, TAGLN and ImmuneScore were − 0.976 (p.value = 0), -1 (p.value = 0), -0.738 (p.value = 0.037), -1 (p.value = 0) (Supplementary Fig. 1). Firstly, VCL had the best AUC values in GSE120521 and GSE41571, both exceeding 0.96. Secondly, VCL had the highest values for all three parameters in the topology analysis of the PPI network. Based on these two points, VCL was selected for subsequent analysis in this study.

**Fig. 6:**
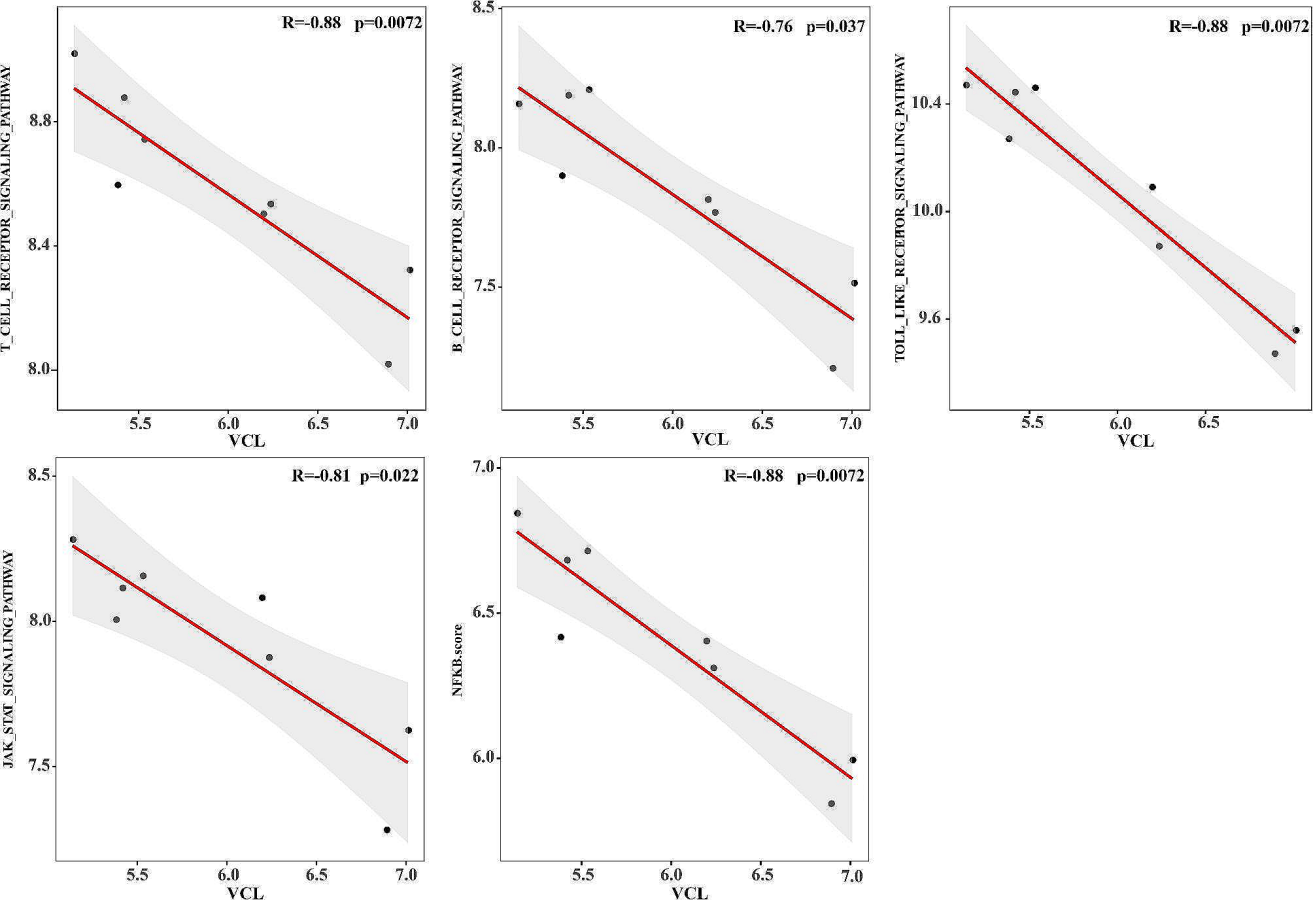
VCL is negatively correlated with pathways. Scatterplot of correlation with VCL expression with AS-related inflammatory pathways. The horizontal coordinate was the expression level of VCL. Vertical coordinates are the ssGSEA scores of T_CELL_RECEPTOR_SIGNALING_PATHWAY, B_CELL_RECEPTOR_SIGNALING_PATHWAY, TOLL_LIKE_RECEPTOR_SIGNALING_PATHWAY, JAK_STAT_SIGNALING_ PATHWAY, and NFKB.score

**Fig. 7:**
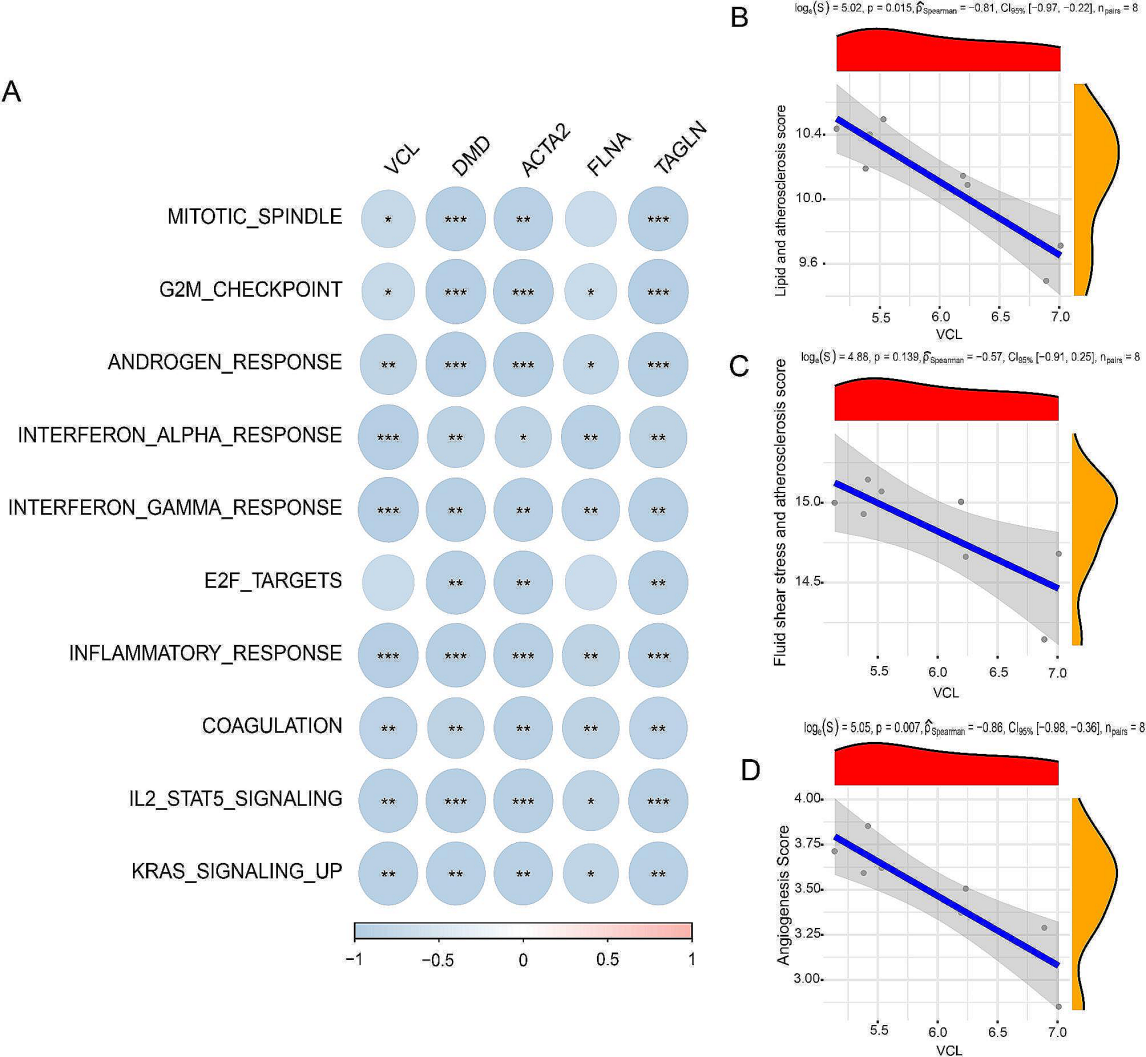
VCL is negatively correlated with pathways. A, VCL is negatively correlated with differential pathways between stable and unstable plaques. B-D, VCL has negative correlation with is AS-related pathways and angiogenesis

Next, we observed VCL was negatively correlated with AS-related inflammatory pathways including T_CELL_RECEPTOR_SIGNALING_PATHWAY (*R*=-0.88, *P* = 0.0072), B_CELL_RECEPTOR_SIGNALING_PATHWAY (*R*=-0.76, *P* = 0.037), TOLL_LIKE_RECEPTOR_SIGNALING_PATHWAY (*R*=-0.88, *P* = 0.0072), JAK_STAT_SIGNALING_PATHWAY (*R*=-0.81, *P* = 0.022), and NF-KB_SIGNALING_PATHWAY (*R*=-0.88, *P* = 0.0072) (Fig. 6). Figure 7A also revealed a negative correlation between VCL and differential pathways between stable and unstable plaques. Additionally, VCL was significantly negatively associated with lipid and atherosclerosis score (*R*=-0.81, *P* = 0.015) and angiogenesis (*R*=-0.86, *P* = 0.007) (Fig. 7B-D).

### Prediction of VCL-related gene sets and potential target drugs

**Fig. 8:**
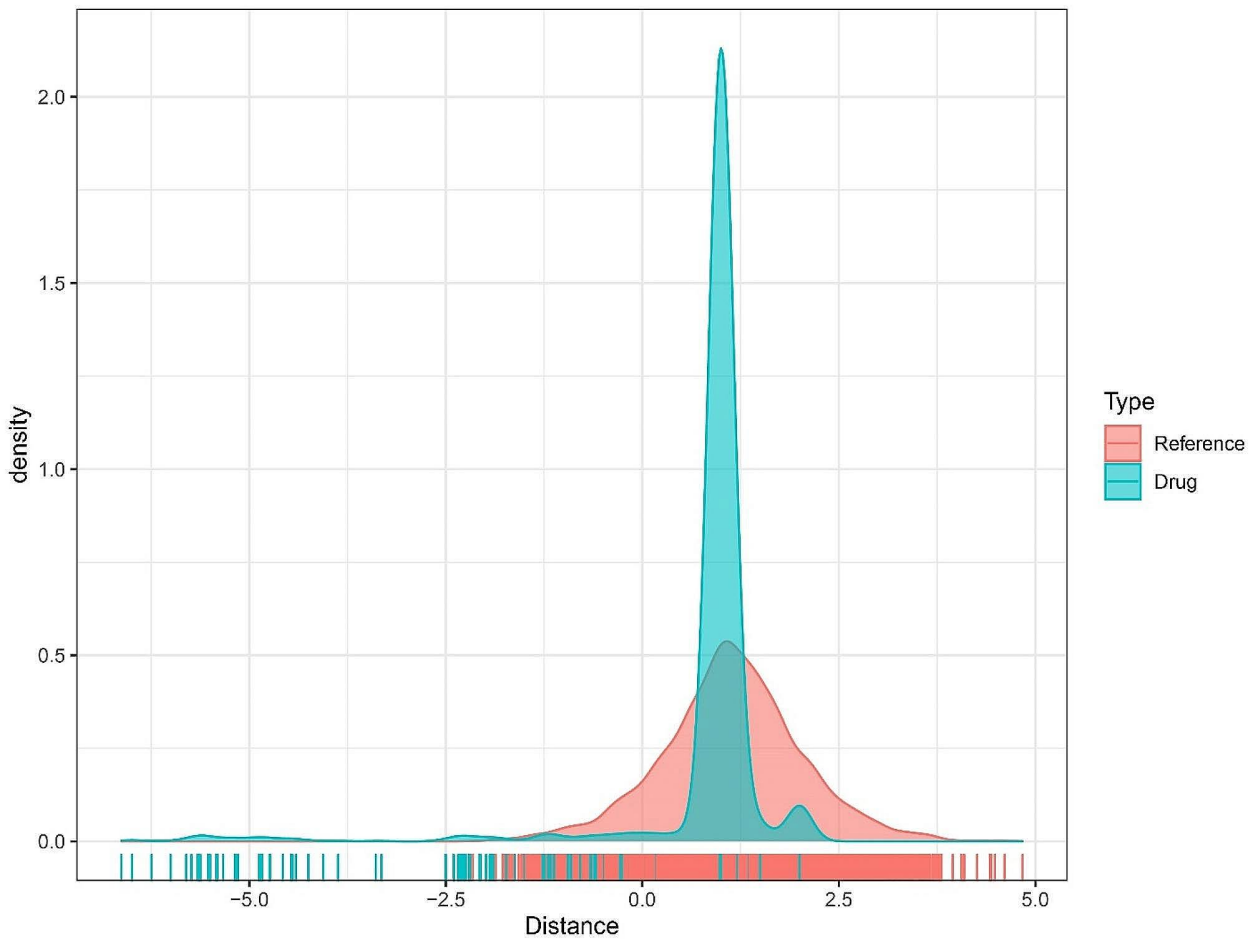
Distance density diagram from drug to VCL-related gene set. The true therapeutic and predictive drug distributions range from − 7.5 to 5.0. Both peak at around 1.2. There was a significant downward trend in the density between the real treatment drug and the predicted drug around 1.2, but the downward trend in the density of the real drug was smoother

Furthermore, we analyzed the correlation between VCL and genes with correlation greater than 0.9 and *P* < 0.001, and we identified 408 genes that were highly correlated with VCL. Subsequently, we calculated the proximity of potential target drugs of VCL-related genes to AS via formula 1 and converted the observed distance to a standardized scoring via formula 2. Based on random data for multiple hypotheses tests, drugs with short distances and FDR < 0.05 were determined as the set of drug candidates associated with the VCL-related gene set (Fig. 8). From the results, it was clearly observed that the true therapeutic drug and predicted drug distributions ranged from − 7.5 to 5.0. The peak was reached near 1.2. The density between the real therapeutic and predicted drugs shows a significant decreasing trend around 1.2, but the decreasing trend of the density of the real drugs is smoother. Therefore, drugs with a distance less than 1.2 may be available for the treatment of AS. therefore 1.2 was chosen as the threshold for drug screening and combining the p-value and FDR value, 152 drugs were considered to have therapeutic potential for AS (Supplementary Table 1).

### Molecular docking and MD simulation

**Fig. 9:**
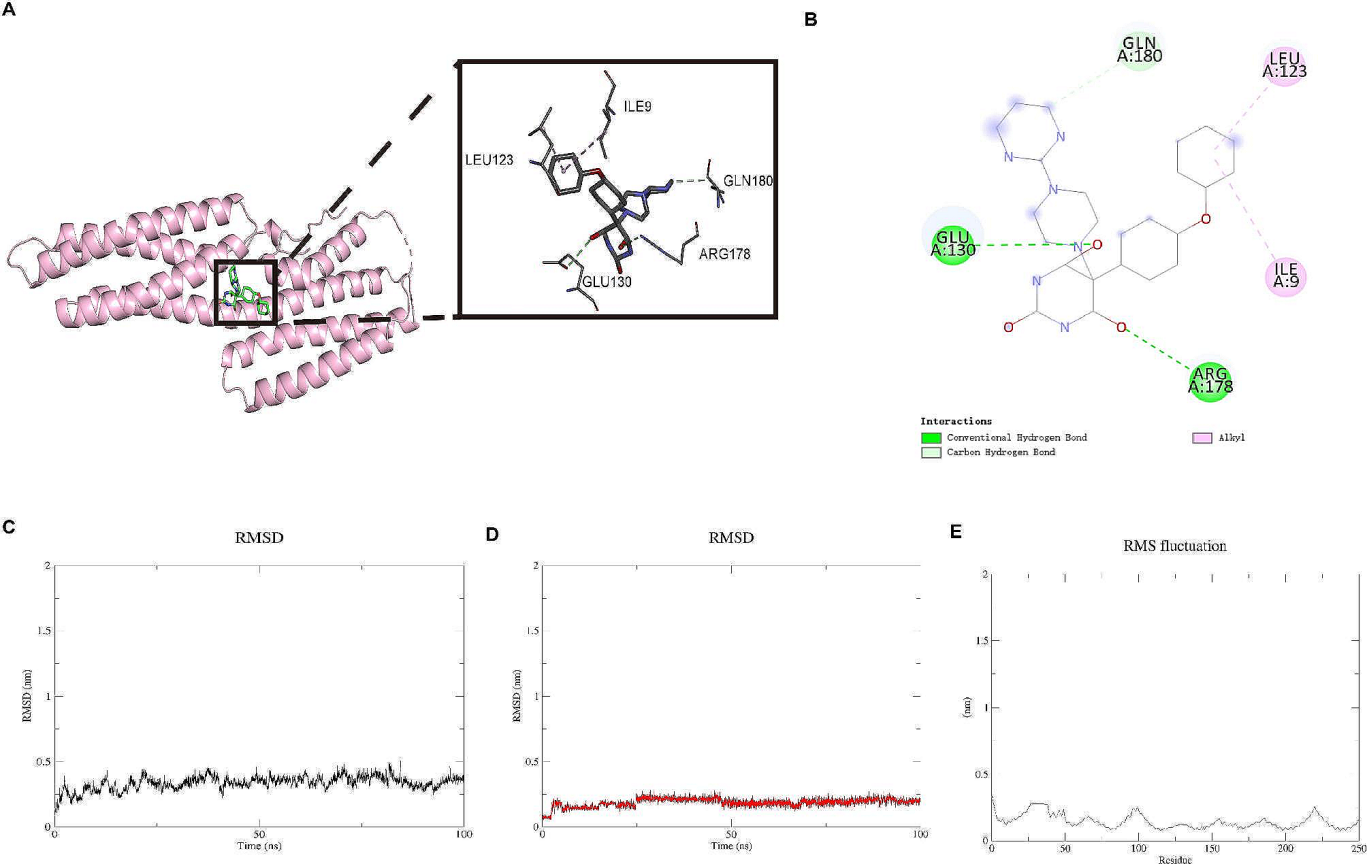
Molecular docking and MD simulations. A, Detailed interaction between compound DB07117 and VCL protein. Light blue bands are protein skeleton, colored sticks represent compound DB07117, and light gray sticks represent the amino acid residues that produce the interaction. The colors of the heteroatoms in the compound and amino acid residues are shown by element types. B, 2D interaction diagram of compound DB07117 with VCL protein. Green dotted line represents the hydrogen bond, light green represents the hydrocarbon bond, magenta dotted line represents the charge attraction, and pink dotted line represents Alkyl interaction. C, The alterations of RMSD values of VCL protein skeleton in the process of 100 ns MD simulation D, The alterations of RMSD values of DB07117 skeleton during 100 ns MD simulation. E, The changes of RMSF values of VCL protein skeleton during 100 ns MD simulation

In molecular docking analysis, binding energy scores less than − 7 kcal/mol were considered to have a strong binding effect. According to the results from molecular docking, four compounds including DB07117, DB05495, DB03683, and DB01949 were predicted (Table 2), and DB07117 has the strongest molecular docking score of -7.7 kcal/mol (Fig. 9A). The compound DB07117 was capable of hydrogen bonding interactions with GLU130 and ARG178, while it had hydrophobic interactions with GLN180, LEU123, and ILE9 (Fig. 9B). Figure 9C displayed the alterations of RMSD values of VCL protein skeleton. The results showed that the conformation of VCL protein was stable in the process of 100 ns MD simulation. Figure 9D depicted the stable RMSD values of compound DB07117, indicating that compound DB07117 and VCL were stably combined. Next, the changes of RMSF values of protein skeleton of VCL during 100 ns MD simulation were further evaluated. Figure 9E showed the VCL protein skeleton was extremely stable. Collectively, compound DB07117 combined with VCL protein stably, which indicated that compound DB07117 was a potential inhibitor of VCL protein.

**Table 2:** The candidate drugs targeting to VCL-related genes

Compounds	Score	H-Bond Interactions	Hydrophobic Interactions
DB07117	-7.7	GLU130, ARG178	GLN180, LEU123, ILE9
DB05495	-6.7		TYR144, LEU164, ILE141
DB03683	-3.9		ILE134, MET174, ARG178
DB01949	-3.7		ILE134, MET174, PHE126

## Discussion

As a progressive condition, AS has been considered as one of the major leading cause of incidence and death worldwide. It is essential to mine innovative biomarkers that can reduce the instability of atherosclerotic plaques and prevent acute cardiovascular events occurrence. In this study, we screened DEGs between unstable plaques and stable plaques and constructed a PPI network to identify 5 hub genes (VCL, DMD, ACTA2, FLNA, and TAGLN). Among them, VCL was identified a key gene that was negatively correlated with immune characteristics and inflammatory pathways. Next, Molecular docking and MD simulation confirmed compound DB07117 was a potential inhibitor of VCL protein, which provided a basis for drug discovery for atherosclerotic plaques.

VCL is a cytoskeletal protein that promotes the adhesions of cell-matrix or cell-cell to actin-based cytoskeleton [38]. It has been reported that specific removal of VCL gene impairs cellular junctions of cardiomyocytes, resulting in sudden death or dilated cardiomyopathy [39]. The Tampere Vascular Study has quantified the expression level of VCL and found that the level of VCL is significantly decreased in both blood samples and atherosclerotic plaques [40]. Additionally, Zhang and colleagues have identified the innovative biomarkers between normal and atherosclerotic arterial endothelial cells, and confirmed that VCL is downregulated in atherosclerotic aortic endothelial, which indicates the role of VCL in the development of AS [41]. Moreover, the apolipoprotein E-deficient (ApoE−/−) mice exhibit highly expressed S721-non-phosphorylatable VCL mutant in endothelial cells. This finding reveals that the phosphorylation of VCL^S721^ damages vascular endothelial junctions and promotes AS development, which suggests that endothelial VCL^S721p^ represents a novel marker for clinical evaluation and treatment of AS-induced vascular diseases [42]. These findings imply that VCL as a focal adhesion protein that is required for the initiation and progression of AS. In this study, we noticed that AS-related DEGs were mainly enriched in focal adhesion, regulation of actin cytoskeleton and ECM-receptor interaction, which demonstrated the role of focal adhesion in the instability of atherosclerotic plaques.

A large body of studies have deciphered that both innate and adaptive immune cells exert pro-inflammatory or anti-inflammatory effects, therefore leading to progression or inhibition of AS [1]. It is assumed that atherosclerotic plaque has autoimmune response by harboring T and B cells. Compared with asymptomatic AS patients, the percentage of macrophages and T lymphocytes in symptomatic AS patients is higher. T lymphocytes enhance the instability of atherosclerotic plaques via the recruitment of macrophages secreting matrix metalloproteinases through CD40 [43]. Depuydt and colleagues have demonstrated there is distinctive clonal proliferation of CD4 + T cells within plaque that expressing CD69, FOS and FOSB, implying the binding of TCRs and antigen-specific stimulation [44]. The activation of T cells obtained from atherosclerotic plaques is induced by the conjugation of malondialdehyde with albumin exhibits pro-inflammatory response [45]. Moreover, B cells secrete various cytokines that significantly affect inflammation and the interplay between B cells and CD4 T cells enhances the progression of AS through the major histocompatibility complex (MHC) II and CD40 [46]. These findings conclude that specific antigens drive an immune response within the atherosclerotic plaques. In this study, we first noticed that VCL was negatively correlated with immune score, T cell receptor signaling pathway and B cell receptor signaling pathway, indicating the regulation of VCL in immune response in atherosclerotic plaques.

Increasing experimental and clinical evidences have revealed that AS is a chronic inflammatory disorder. Inflammation has been proven to link dyslipidaemia and other risk contributors to AS and inflammatory mediators regulate many aspects of disruption and healing process of plaques [47]. Inflammatory cells such as T-lymphocytes, dendritic cells and macrophages have been observed within plaques and contribute to the instability of plaques [48]. Under condition of hypoxia and ischemia, the activation of NF-κB pathway may provoke pathological events including innate and adaptive immunity, and cell survival, differentiation, and proliferation [49]. A previous experimental study has demonstrated that inhibition of p38 MAPK/NF-κB pathway suppresses the progression of AS [50]. A recent study has revealed that activating the JAK2-STAT2 pathway via the membrane translocation of FABP4 and the phosphorylation of c-Src enhances macrophage inflammation in ApoE−/− mice [51]. However, inhibition of JAK-STAT pathway can attenuate inflammatory response in AS [52, 53]. Additionally, toll-like receptors (TLRs) have been considered as crucial mediators of atherosclerotic diseases. Their activations promote MyD88 or TRIF-induced intracellular signaling cascade, which results in the release of pro- or anti-inflammatory cytokines [54]. TLR2 and TLR4 deficiencies markedly impair innate immune signaling and reduce oral bacteria-induced AS [55]. These findings suggest that the NF-κB pathway, JAK-STAT pathway and TLRs pathway sustains inflammatory response and therefore results in AS, while regulation of these pathway may represent a potential strategy for AS. Our study had demonstrated a negative correlation between VCL and these inflammatory pathways, which might unveil the underlying mechanism of VCL in the instability of atherosclerotic plaques.

Molecular docking is an important method for drug discovery and has become a core computational approach in drug design to predict the binding affinity and provide the interactive mode [56]. In recent years, molecular docking combined with MD simulation has been utilized to identify the molecular targets of several potential major bioactive components for AS including punicalagin, quercetin and luteolin, which provide a basis for pharmacological research and clinical application for AS [57–59]. In the current study, we identified compound DB07117 combined with VCL protein stably, which indicated that compound DB07117 was a potential inhibitor of VCL protein.

However, some limitations should be addressed in this study. We downloaded retrospective data of GSE120521 and GSE41571 from the GEO with small sample size and this may lead to bias. Therefore, further prospective studies with large sample size should be carried out to avoid the bias. Although we have validated the results in GSE41571 dataset, the diagnostic value of this model should be validated in dataset containing follow-up collection of clinical samples. Additionally, we have identified VCL as the key gene that was negatively associated with immune characteristics and pathways, the potential mechanism of VCL should be verified by functional experimental studies.

## Conclusion

In conclusion, we screened 5 hub genes and constructed a diagnostic model that had a good performance in diagnostic prediction. Among the 5 hub genes, VCL was identified as a key gene that was negatively associated with immune score and inflammatory pathways. Molecular docking and MD simulation confirmed compound DB07117 was a potential inhibitor of VCL protein. This finding provided a promising therapeutic biomarker for the treatment of atherosclerotic plaques.

### Electronic supplementary material

Below is the link to the electronic supplementary material.

Supplementary Material 1

Supplementary Material 2

Supplementary Material 3

Supplementary Material 4

## Data Availability

Experimental data collected and analyzed in this research are available from the first author and the corresponding author upon request.

## Abbreviations

AS: Atherosclerosis
GEO: Gene-Expression Omnibus
DEGs: Differential expressed genes
MD: Molecular dynamics
FDR: False discovery rate
GO: Gene ontology
BP: Biological process
CC: Cellular component
MF: Molecular function
PPI: Protein–protein interactions
MCODE: Molecular complex detection
SVM: Support vector machine
ssGSEA: Single-sample gene set enrichment analysis
ROC: Receiver operating characteristic analysis
AUC: Area under ROC curve
PDB: Protein Data Bank
CGenFF: CHARMM Common Force Field
PME: Particle-Mesh Ewald
RMSD: Root mean square deviation
NVT: Canonical ensemble
NPT: Isobaric-isothermal ensemble
MHC: Major histocompatibility complex
VCL: Vinculin
DMD: Dystrophin
ACTA2: Actin alpha 2
FLNA: Filamin A

## References

[1] Wolf D, Ley K (2019). Immunity and inflammation in atherosclerosis. Circul Res.

[2] Wengrofsky P, Lee J, Makaryus AN (2019). Dyslipidemia and its role in the pathogenesis of atherosclerotic cardiovascular disease: implications for evaluation and targets for treatment of dyslipidemia based on recent guidelines.

[3] Libby P (2021). The changing nature of atherosclerosis: what we thought we knew, what we think we know, and what we have to learn. Eur Heart J.

[4] Song P, Fang Z, Wang H, Cai Y, Rahimi K, Zhu Y (2020). Global and regional prevalence, burden, and risk factors for carotid atherosclerosis: a systematic review, meta-analysis, and modelling study. The Lancet Global Health.

[5] Vergallo R, Crea F (2020). Atherosclerotic plaque healing. N Engl J Med.

[6] Libby P (2021). The changing landscape of atherosclerosis. Nature.

[7] Bozaykut P, Ekren R, Sezerman OU, Gladyshev VN, Ozer NK (2020). High-throughput profiling reveals perturbation of endoplasmic reticulum stress‐related genes in atherosclerosis induced by high‐cholesterol diet and the protective role of vitamin E. BioFactors.

[8] Eberhardt N, Giannarelli C, Arteriosclerosis (2022). Thromb Vascular Biology.

[9] Athanasios A, Charalampos V, Vasileios T (2017). Protein-protein interaction (PPI) network: recent advances in drug discovery. Curr Drug Metab.

[10] Yu H, Kim PM, Sprecher E, Trifonov V, Gerstein M (2007). The importance of bottlenecks in protein networks: correlation with gene essentiality and expression dynamics. PLoS Comput Biol.

[11] Santos LH, Ferreira RS, Caffarena ER. Integrating molecular docking and molecular dynamics simulations. Docking screens for drug discovery. 2019:13–34. 10.1007/978-1-4939-9752-7_2 31452096

[12] Singh S, Baker QB, Singh DB (2022). Molecular docking and molecular dynamics simulation.

[13] Liu Y, Huan W, Wu J, Zou S, Qu L (2020). IGFBP6 is downregulated in unstable carotid atherosclerotic plaques according to an integrated bioinformatics analysis and experimental verification. J Atheroscler Thromb.

[14] Guo J, Ning Y, Su Z, Guo L, Gu Y (2022). Identification of hub genes and regulatory networks in histologically unstable carotid atherosclerotic plaque by bioinformatics analysis. BMC Med Genom.

[15] Xu X, Zhang Y, Lu X, Shi B. Combining network pharmacology and bioinformatics analysis to identify the molecular mechanisms of UDCA in the treatment of carotid atherosclerosis. 2022.

[16] Ritchie ME, Phipson B, Wu D, Hu Y, Law CW, Shi W (2015). Limma powers differential expression analyses for RNA-sequencing and microarray studies. Nucleic Acids Res.

[17] Liao Y, Wang J, Jaehnig EJ, Shi Z, Zhang B (2019). WebGestalt 2019: gene set analysis toolkit with revamped UIs and APIs. Nucleic Acids Res.

[18] Shannon P, Markiel A, Ozier O, Baliga NS, Wang JT, Ramage D (2003). Cytoscape: a software environment for integrated models of biomolecular interaction networks. Genome Res.

[19] Meyer D, Dimitriadou E, Hornik K, Weingessel A, Leisch F, Chang C-C et al. Package ‘e1071’. R J. 2019.

[20] Charoentong P, Finotello F, Angelova M, Mayer C, Efremova M, Rieder D (2017). Pan-cancer immunogenomic analyses reveal genotype-immunophenotype relationships and predictors of response to checkpoint blockade. Cell Rep.

[21] Barbie DA, Tamayo P, Boehm JS, Kim SY, Moody SE, Dunn IF (2009). Systematic RNA interference reveals that oncogenic KRAS-driven cancers require TBK1. Nature.

[22] Yoshihara K, Shahmoradgoli M, Martinez E, Vegesna R, Kim H, Torres-Garcia W (2013). Inferring tumour purity and stromal and immune cell admixture from expression data. Nat Commun.

[23] Zhu Y, Xian X, Wang Z, Bi Y, Chen Q, Han X (2018). Research progress on the relationship between atherosclerosis and inflammation. Biomolecules.

[24] Harrell FE Jr, Harrell MFE Jr. Package ‘hmisc’. CRAN2018. 2019;2019:235-6.

[25] Peng Y, Yuan M, Xin J, Liu X, Wang J (2020). Screening novel drug candidates for Alzheimer’s disease by an integrated network and transcriptome analysis. Bioinformatics.

[26] Franceschini A, Szklarczyk MD, RUnit S. biocViews Network B. Package ‘STRINGdb’. 2015.

[27] Jiménez J, Doerr S, Martínez-Rosell G, Rose AS, De Fabritiis G (2017). DeepSite: protein-binding site predictor using 3D-convolutional neural networks. Bioinformatics.

[28] Trott O, Olson AJ (2010). AutoDock Vina: improving the speed and accuracy of docking with a new scoring function, efficient optimization, and multithreading. J Comput Chem.

[29] El-Hachem N, Haibe-Kains B, Khalil A, Kobeissy FH, Nemer G. AutoDock and AutoDockTools for protein-ligand docking: beta-site amyloid precursor protein cleaving enzyme 1 (BACE1) as a case study. Neuroproteomics: Methods and Protocols. 2017:391–403. 10.1007/978-1-4939-6952-4_20 28508374

[30] DeLano WL, Pymol (2002). An open-source molecular graphics tool. CCP4 Newsl Protein Crystallogr.

[31] Abraham MJ, Murtola T, Schulz R, Páll S, Smith JC, Hess B (2015). GROMACS: high performance molecular simulations through multi-level parallelism from laptops to supercomputers. SoftwareX.

[32] Zhu X, Lopes PE, Mackerell AD (2012). Jr. Recent developments and applications of the CHARMM force fields. Wiley Interdiscip Rev Comput Mol Sci.

[33] Vanommeslaeghe K, MacKerell AD (2012). Automation of the CHARMM General Force Field (CGenFF) I: bond perception and atom typing. J Chem Inf Model.

[34] Vanommeslaeghe K, Raman EP, MacKerell AD (2012). Automation of the CHARMM General Force Field (CGenFF) II: assignment of bonded parameters and partial atomic charges. J Chem Inf Model.

[35] Hess B, Bekker H, Berendsen HJ, Fraaije JG (1997). LINCS: a linear constraint solver for molecular simulations. J Comput Chem.

[36] Darden T, Perera L, Li L, Pedersen L (1999). New tricks for modelers from the crystallography toolkit: the particle mesh Ewald algorithm and its use in nucleic acid simulations. Structure.

[37] Van Der Spoel D, Lindahl E, Hess B, Groenhof G, Mark AE, Berendsen HJ (2005). GROMACS: fast, flexible, and free. J Comput Chem.

[38] Mandal P, Belapurkar V, Nair D, Ramanan N (2021). Vinculin-mediated axon growth requires interaction with actin but not talin in mouse neocortical neurons. Cell Mol Life Sci.

[39] Zemljic-Harpf AE, Miller JC, Henderson SA, Wright AT, Manso AM, Elsherif L (2007). Cardiac-myocyte-specific excision of the vinculin gene disrupts cellular junctions, causing sudden death or dilated cardiomyopathy. Mol Cell Biol.

[40] von Essen M, Rahikainen R, Oksala N, Raitoharju E, Seppälä I, Mennander A (2016). Talin and vinculin are downregulated in atherosclerotic plaque; Tampere Vascular Study. Atherosclerosis.

[41] Zhang W, Jianping W, Dong J, Wenwen L, Wang X, Hou Y. CAV1 and VCL are Downregulated in Atherosclerotic Aortic Endothelial. 2021.

[42] Shih Y-T, Wei S-Y, Chen J-H, Wang W-L, Wu H-Y, Wang M-C (2023). Vinculin phosphorylation impairs vascular endothelial junctions promoting atherosclerosis. Eur Heart J.

[43] Mughal MM, Khan MK, DeMarco JK, Majid A, Shamoun F, Abela GS (2011). Symptomatic and asymptomatic carotid artery plaque. Expert Rev Cardiovasc Ther.

[44] Depuydt MA, Schaftenaar FH, Prange KH, Boltjes A, Hemme E, Delfos L et al. Single-cell T cell receptor sequencing of paired human atherosclerotic plaques and blood reveals autoimmune-like features of expanded effector T cells. Nat Cardiovasc Res. 2023:1–14. 10.1038/s44161-022-00208-4 PMC11041750 38665903

[45] Rahman M, Steuer J, Gillgren P, Végvári Á, Liu A, Frostegård J (2019). Malondialdehyde conjugated with albumin induces pro-inflammatory activation of T cells isolated from human atherosclerotic plaques both directly and via dendritic cell–mediated mechanism. JACC: Basic to Translational Science.

[46] Tay C, Kanellakis P, Hosseini H, Cao A, Toh B-H, Bobik A (2020). B cell and CD4 T cell interactions promote development of atherosclerosis. Front Immunol.

[47] Libby P (2021). Inflammation during the life cycle of the atherosclerotic plaque. Cardiovascular Res.

[48] Rai V, Rao VH, Shao Z, Agrawal DK (2016). Dendritic cells expressing triggering receptor expressed on myeloid cells-1 correlate with plaque stability in symptomatic and asymptomatic patients with carotid stenosis. PLoS ONE.

[49] Cheng W, Cui C, Liu G, Ye C, Shao F, Bagchi AK et al. NF-κB, a potential therapeutic target in cardiovascular diseases. Cardiovasc Drugs Ther. 2022:1–14. 10.1007/s10557-022-07362-8 35796905

[50] Lima GF, de Oliveira Lopes R, Mendes ABA, Brazão SC, Autran LJ, Motta NAV (2020). Inosine, an endogenous purine nucleoside, avoids early stages of atherosclerosis development associated to eNOS activation and p38 MAPK/NF-kB inhibition in rats. Eur J Pharmacol.

[51] Xu L, Zhang H, Wang Y, Yang A, Dong X, Gu L (2022). FABP4 activates the JAK2/STAT2 pathway via Rap1a in the homocysteine-induced macrophage inflammatory response in ApoE–/– mice atherosclerosis. Lab Invest.

[52] Fu X, Sun Z, Long Q, Tan W, Ding H, Liu X (2022). Glycosides from Buyang Huanwu Decoction inhibit atherosclerotic inflammation via JAK/STAT signaling pathway. Phytomedicine.

[53] Yang X, Jia J, Yu Z, Duanmu Z, He H, Chen S (2020). Inhibition of JAK2/STAT3/SOCS3 signaling attenuates atherosclerosis in rabbit. BMC Cardiovasc Disord.

[54] Falck-Hansen M, Kassiteridi C, Monaco C (2013). Toll-like receptors in atherosclerosis. Int J Mol Sci.

[55] Chukkapalli SS, Ambadapadi S, Varkoly K, Jiron J, Aguirre JI, Bhattacharyya I (2018). Impaired innate immune signaling due to combined toll-like receptor 2 and 4 deficiency affects both periodontitis and atherosclerosis in response to polybacterial infection. Pathogens and Disease.

[56] Fan J, Fu A, Zhang L (2019). Progress in molecular docking. Quant Biology.

[57] Lee AY, Lee J-Y, Chun JM (2020). Exploring the mechanism of Gyejibokryeong-Hwan against atherosclerosis using network pharmacology and molecular docking. Plants.

[58] Zhu R, Du B, Yuan J, Yan S, Shao M, Sang F et al. Potential mechanisms of Biejiajian Pill in the treatment of diabetic atherosclerosis based on network pharmacology, molecular docking, and molecular dynamics simulation. Evidence-Based Complementary and Alternative Medicine. 2022;2022. 10.1155/2022/3296279 PMC9391107 35990823

[59] Huwait E, Almowallad S, Al-Massabi R, Saddeek S, Gauthaman K, Prola A (2022). Punicalagin targets atherosclerosis: gene expression profiling of THP-1 macrophages treated with Punicalagin and Molecular Docking. Curr Issues Mol Biol.

