# Supplementary figures and images for: Identification of a hub gene VCL for atherosclerotic plaques and discovery of potential therapeutic targets by molecular docking
Source: BMC Med Genomics. 2024 Jan 29;17:42. doi: 10.1186/s12920-024-01815-9 (PMC10826019; doi:10.1186/s12920-024-01815-9)

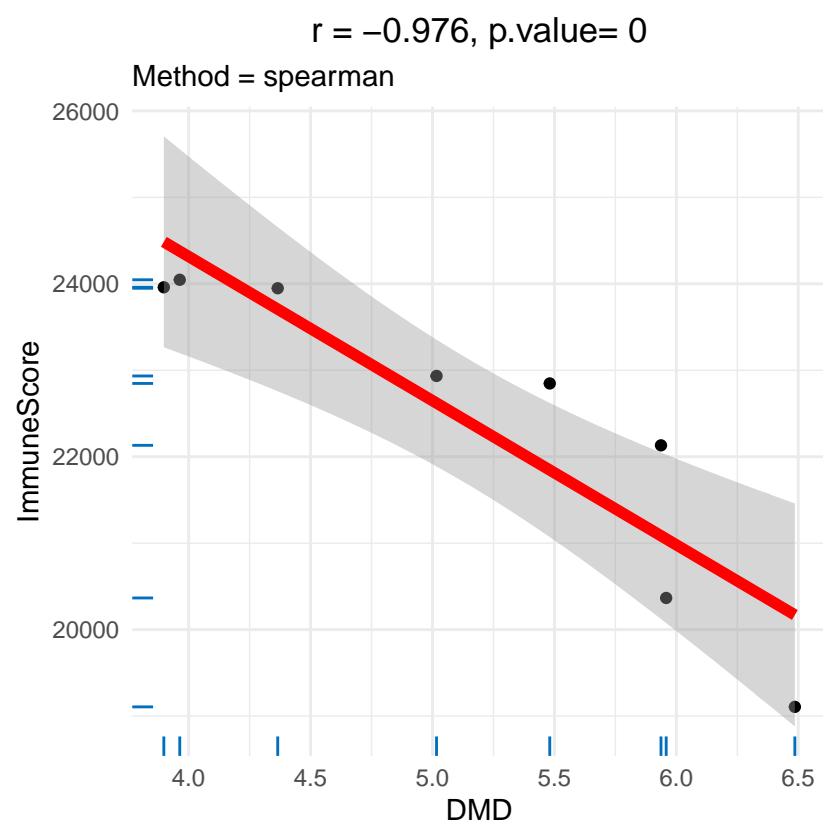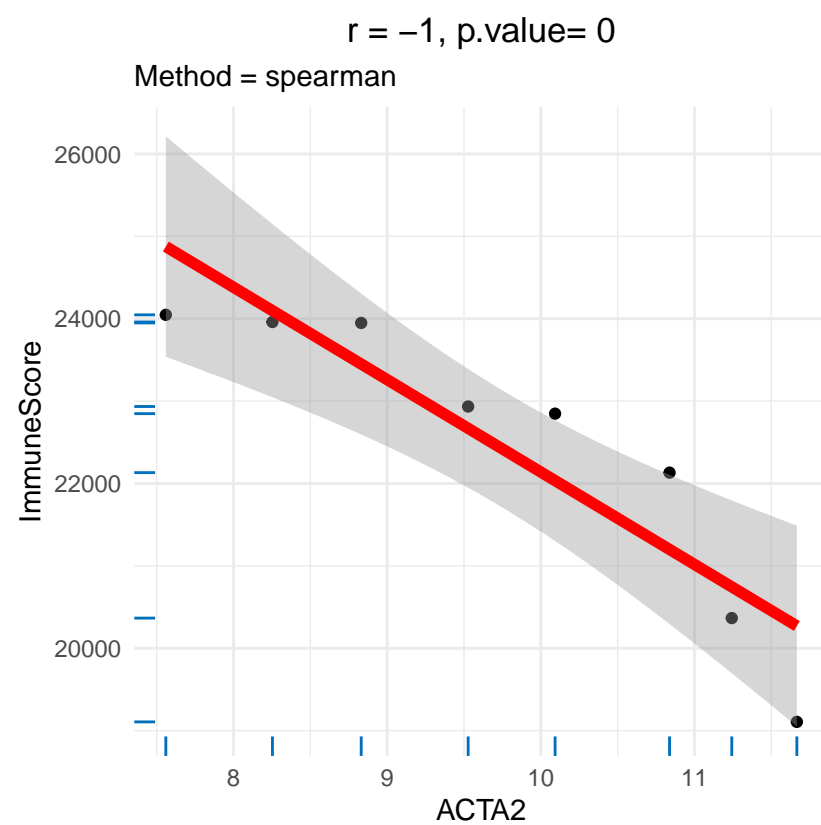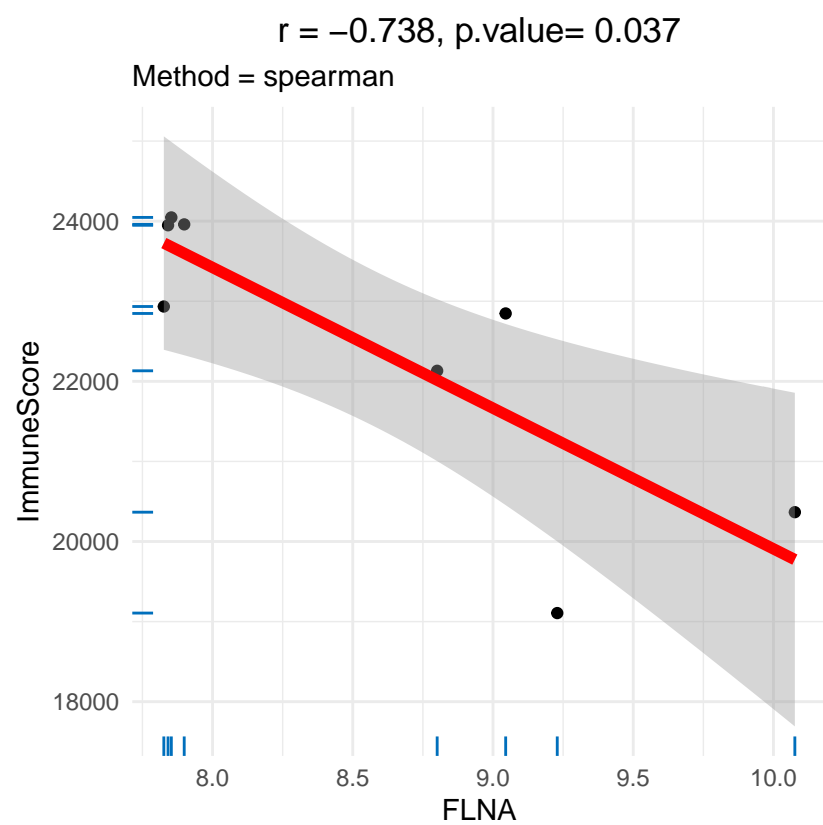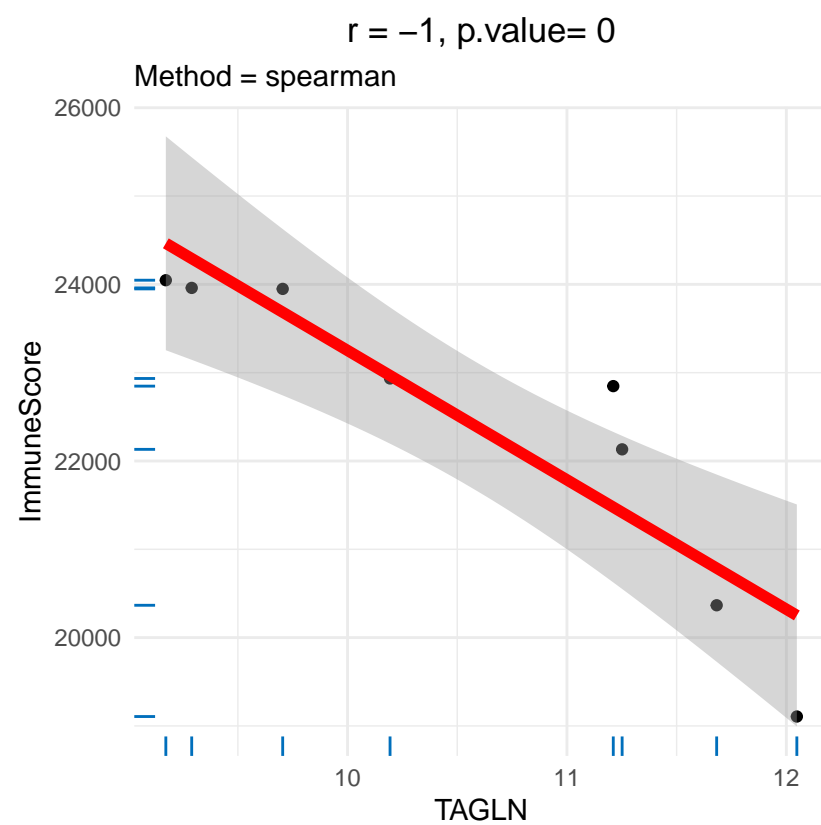

Supplement: Supplementary file 2 — Supplementary Material 2 [file 12920_2024_1815_MOESM2_ESM.pdf]
