# Supplementary material for: Identification of a hub gene VCL for atherosclerotic plaques and discovery of potential therapeutic targets by molecular docking
Source: BMC Med Genomics. 2024 Jan 29;17:42. doi: 10.1186/s12920-024-01815-9 (PMC10826019; doi:10.1186/s12920-024-01815-9)
Supplement: Supplementary file 3 — Supplementary Material 3 [file 12920_2024_1815_MOESM3_ESM.docx]

Supplementary Table 1, Predicted AS therapeutic agents based on PPI network of VCL-related genes.
