# Supplementary material for: Identification of a hub gene VCL for atherosclerotic plaques and discovery of potential therapeutic targets by molecular docking
Source: BMC Med Genomics. 2024 Jan 29;17:42. doi: 10.1186/s12920-024-01815-9 (PMC10826019; doi:10.1186/s12920-024-01815-9)
Supplement: Supplementary file 4 — Supplementary Material 4 [file 12920_2024_1815_MOESM4_ESM.docx]

**Supplementary Figure 1** Correlation of DMD, ACTA2, FLNA, and TAGLN with immune scores.
